# Under-5 mortality surveillance in low-income and middle-income countries: insights from two Health and Demographic Surveillance Systems in rural Gambia

**DOI:** 10.1136/bmjgh-2023-014937

**Published:** 2024-04-02

**Authors:** Baleng Mahama Wutor, Isaac Osei, Golam Sarwar, Williams Oluwatosin Adefila, Lobga Babila Galega, Ilias Hossain, Yusuf Abdulsalam, Keita Modou Lamin, Alhagie Muya Baldeh, Basiru Barry, Esu Ezeani, Grant Mackenzie

**Affiliations:** 1 Disease Control and Elimination, Medical Research Council Unit The Gambia at the London School of Hygiene and Tropical Medicine, Banjul, Gambia; 2 Faculty of Infectious and Tropical Diseases, London School of Hygiene and Tropical Medicine, London, UK; 3 Murdoch Children’s Research Institute, Melbourne, Victoria, Australia; 4 Department of Paediatrics, University of Melbourne, Melbourne, Victoria, Australia

**Keywords:** child health, global health

## Abstract

Without complete data on under-5 mortality, tracking progress towards achieving Sustainable Development Goal 3.2 will be challenging. Such data are also needed to ensure proper planning and prioritisation of scarce resources in low-income and middle-income countries. However, most low-income and middle-income countries have weak Civil Registration and Vital Statistics (CRVS) systems, leaving a critical gap in understanding under-5 mortality dynamics. This paper outlines a community-based approach to enhance under-5 mortality surveillance in low-income countries, using The Gambia as a case study. The methodology involves Health and Demographic Surveillance Systems (HDSSs) in Basse and Fuladu West, employing unique identification numbers, periodical household visits and collaboration with communities, village reporters and project field workers to ensure comprehensive data collection. Verbal autopsies (VAs) are conducted by trained field workers, and causes of death are determined using the physician-certified VA method. Between 1 September 2019 and 1 September 2023, 1333 deaths were detected, for which causes of death were determined for 97.1% (1294 of 1333). The most common causes of death detected were acute respiratory infections including pneumonia, sepsis, diarrhoeal diseases and birth asphyxia. Challenges include the cost of maintaining the HDSSs, poor road infrastructure, Electronic Data Capture transition challenges, and the need for national integration of HDSS data into the CRVS system. The success of this model highlights its potential for scalable and adaptable under-5 mortality surveillance in resource-limited settings.

**Summary box**

- It is challenging to track progress towards achieving Sustainable Development Goal 3.2, which aims to reduce under-5 mortality to at least 25 per 1000 live births by 2030.
- The main reason for this is the weak Civil Registration and Vital Statistics (CRVS) systems in low-income and middle-income countries; consequently, a significant number of under-5 deaths are not reported, and their causes of death remain a mystery.
- We describe a model for the detection, reporting and determination of causes of death of children under-5 within the context of two Health and Demographic Surveillance Sites in rural Gambia.
- The model’s success in detecting 1333 under-5 deaths and determining the causes of death for 1294 of them over 4 years demonstrates its potential to be replicated in similar low-resource settings as an interim measure until most countries have developed robust CRVS systems.
- Using the physician-certified verbal autopsy method, the leading causes of death were acute respiratory infections including pneumonia, sepsis, diarrhoeal diseases and birth asphyxia.

## Introduction

Even though great progress has been made in reducing under-5 mortality, the number of children not living to celebrate their fifth birthday remains unacceptably high. In 2020, an estimated 5 million children under-5 died.^1^ Most of these deaths are from causes that are preventable and treatable. The inequality in healthcare across the world is reflected in the distribution of under-5 mortality. Over half of under-5 deaths occur in sub-Saharan Africa, and the under-5 mortality rate in this region is 74 deaths per 1000 live births compared with 5 deaths per 1000 live births in Europe.^2 3^ These numbers, however, may be the tip of the iceberg because the low-income and middle-income countries (LMICs) where most of these deaths occur have weak Civil Registration and Vital Statistics (CRVS) systems.^2 4^ A significant number of deaths, especially of children under-5, go unreported. Even for those that are reported, the causes of death remain a mystery.^5 6^ Peter Byass, a pioneer in the determination of causes of death in resource-limited settings, put it aptly when he said that “…the chance of a death being registered and documented as to cause depends strongly on the socioeconomic status of the community and nation in which it occurs.”^7^

Without complete data on under-5 mortality, it would be challenging to plan and allocate resources at the local level. Beyond this, however, the ability to track progress made in achieving Sustainable Development Goal 3.2 relies on robust systems for detecting deaths of children under-5 and determining what resulted in those deaths. Also, donor agencies and global funders rely on such data to set priorities for funding and research projects. We will be missing an essential piece of the puzzle without a system to detect these deaths and find their cause. It will take several years before most countries can develop robust CRVS systems.^8^ In the meantime, innovative approaches must be employed for under-5 mortality surveillance in LMICs.

Where they exist, Health and Demographic Surveillance Systems (HDSSs) collect longitudinal data on pregnancies, births, marriages, deaths and migration within specified populations. HDSSs ensure that almost all deaths within the geographical area are recorded.^9^ The Basse and Fuladu West HDSSs in the eastern part of The Gambia have consistently done this since 2007. However, it is not just enough to identify deaths; it is more useful when the causes of those deaths are determined. In this regard, the WHO and several universities over the years have developed tools to determine causes of death in settings where significant deaths occur at home. These tools often rely on verbal autopsies (VAs) conducted as part of HDSS activities.

A VA is an interview with the primary caregiver of the deceased about the signs and symptoms they exhibited during the period of the illness.^10^ Based on the interviews, computer algorithms or physicians then determine the likely causes of death using International Classification of Diseases (ICD) codes. Computer algorithms such as InterVA, InSilicoVA and SmartVA have been validated to determine causes of death using information from VA interviews.^11 12^ These algorithms provide a cost-effective and logistically feasible method of determining causes of death.

The other way that causes of death have been determined is the use of the physician-certified VA (PCVA) method.^13^ PCVA usually involves two physicians who independently determine causes of death based on the information obtained from VA interviews. The causes of death are compared to determine discordant diagnoses. Where there are disagreements, the two physicians meet to reach a consensus. In some instances, a third physician is called upon to determine the cause of death when the two physicians disagree.

Although VAs are not the most accurate way to determine causes of death, they provide critical information in settings where comprehensive medical records or postmortem examinations are limited.

## Approach to under-5 mortality surveillance

### Setting

The Gambia is a small country in West Africa with a population of 2.2 million. Children under-5 make up 15.4% of the population.^14^ The country is classified by the World Bank as a low-income country with a gross domestic product per capita of US$840.^15^ The public health system in The Gambia operates with limited human and physical resources. Like many developing countries, it has a weak CRVS system. Though the Gambian constitution (Births, Deaths and Marriages Registration Act 1990) places a burden on relatives or neighbours to report deaths that they know of, many deaths still go unreported.^16^ Unlike other age categories, the Act exempts the payment of fees for the registration of deaths of children under-5.

**Figure 1:**
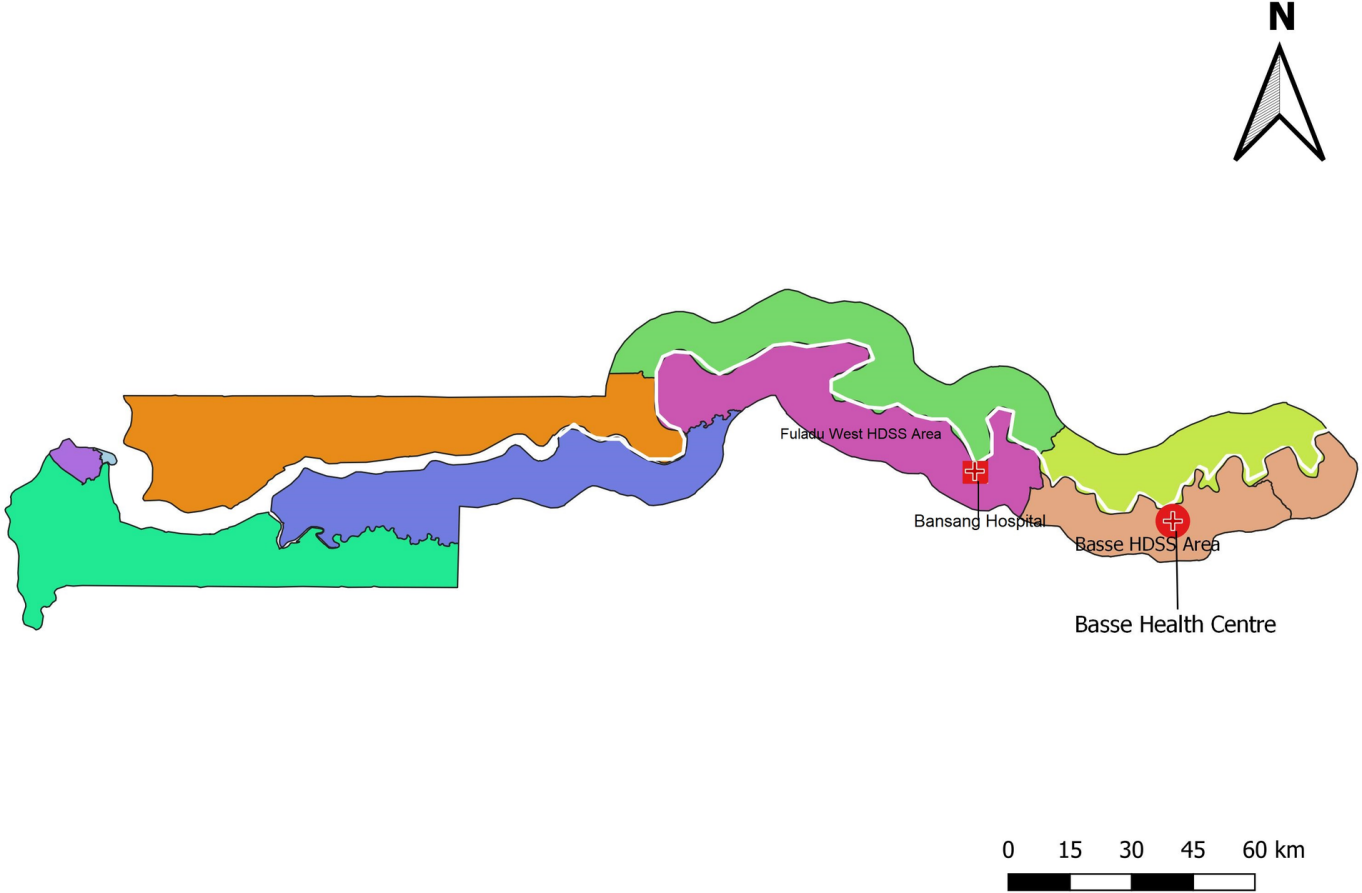
Map of The Gambia highlighting the Basse and Fuladu West Health and Demographic Surveillance Systems (HDSSs).

The Basse HDSS (figure 1) was established by the Medical Research Council Unit The Gambia at the London School of Hygiene and Tropical Medicine (MRCG at LSHTM) in 2007 in the rural eastern part of The Gambia. It covers an area of 1100 km^2^ and has a population of 202 000. Children under-5 make up 19% of the population. The Basse HDSS was set up strategically to cover the southern half of the Upper River Region with the River Gambia serving as a natural boundary of the HDSS. The HDSS was set up to provide accurate estimates of the population denominator for the Pneumococcal Surveillance Project.^17^ Before setting up the HDSS in 2007, an initial census was undertaken over a period of 2 months (November–December). The initial enumerated population in 2006 was 136 387 and included 224 villages and hamlets.^17 18^ The HDSS has been vital in the conduct of several observational studies and clinical trials.^19–21^ The Fuladu West HDSS (figure 1) was set up in 2011 and is contiguous with the Basse HDSS, extending westward towards the coast. The HDSS has been instrumental in supporting the Vaccine Impact on Diarrhoea in Africa Study, the Pneumococcal Surveillance Project and the Pneumococcal Vaccine Schedules (PVS) trial.^17 20 22^ It has a population of 99 113. Given that half of the deaths occurring among children under-5 within the two HDSSs occur at home, death identification through the HDSSs has been an important safety element during studies.^23^ In both HDSSs, a robust system is in place to determine under-5 deaths. This system has relied on a close collaboration between HDSS field workers, project field workers, village reporters (VRs) and community chiefs (‘Alkalos’).

### Death notification within the Basse and Fuladu West Health and Demographic Surveillance Systems

**Figure 2:**
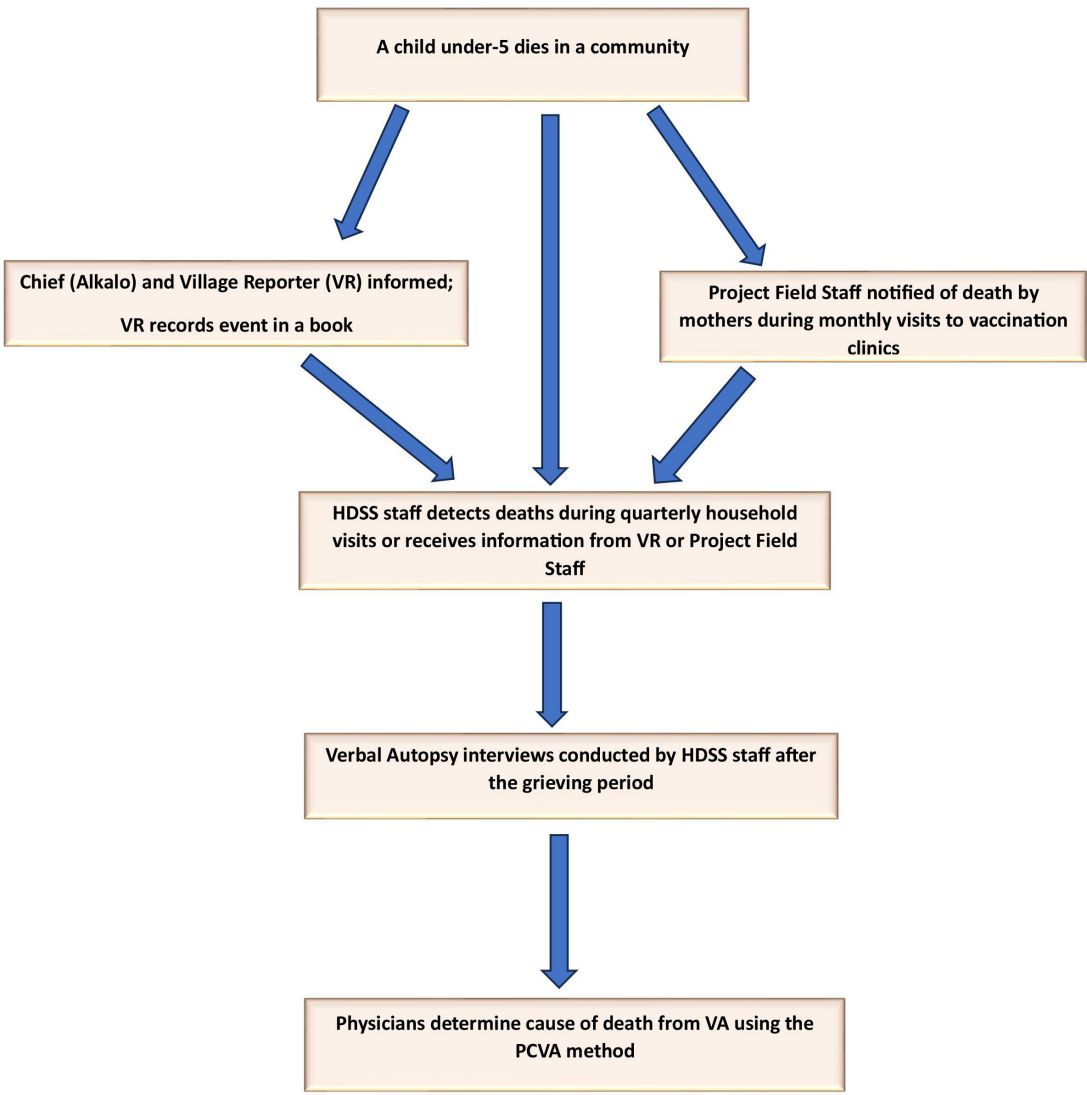
Process flow for under-5 mortality surveillance within the Basse and Fuladu West HDSSs. HDSS, Health and Demographic Surveillance System; PCVA, physician-certified VA; VA, verbal autopsy.

Activities of the Basse HDSS have been described elsewhere.^18^ In both HDSSs, every household member within the geographical area is given a unique 14-digit HDSS identification number which is a combination of their village, compound, household and individual unique number. Trained field workers who usually have a minimum of secondary school education conduct household visits every 4 months to collect health and demographic data. The head of the household is the primary respondent during such visits. However, individual household members are called upon to confirm and update their information within the database. Field workers collect information about new births since the last visit, marriages, migrations and deaths. The household head is asked to confirm the status of each member of the household. Where they indicate that a particular household member has died, the date of death is collected. The information collected at the level of the household is compared with the prospective data collected by resident VRs and any discrepancies resolved with the VRs and household heads. The process flow for detecting and reporting deaths is shown in figure 2. Over the years, the HDSS team has developed cordial relationships with the communities within the study area. Therefore, households rarely decline their visits or requests to conduct VAs. Deaths that have occurred since the last visit are flagged and subsequent VA interviews are scheduled after the required grieving period.

### Village reporters

To ensure the prospective collection of data on births, deaths and migrations, volunteer VRs who are natives and residents within the villages collect data on these events. The VRs are appointed by Alkalos and are often respected people within the village. Some VRs double as community health nurses, enabling them to also capture deaths that occur at the health facilities. The HDSS team trains them to keep records of births, deaths, and migration in and out of the village. Each VR has a designated book in which such information is captured. Field workers compare the information captured in the VR’s book with the data collected during household visits to avoid duplication and to ensure that complete data are collected.

### Project field workers

For the past 4 years, the Pneumococcal Vaccine Schedules (PVS) trial, which seeks to compare an alternative schedule of the pneumococcal conjugate vaccine in infants, has been ongoing within the two HDSSs.^20^ PVS field workers undertake monthly visits to clusters of communities to attend childhood immunisation clinics. During these routine visits, data are collected from mothers, including details of pregnancies, births, vaccinations and deaths. They notify the HDSS team when they detect any deaths of children under-5.

### Conduct of verbal autopsies

Field workers are periodically trained to conduct effective VA interviews using training manuals developed by the WHO.^24^ They are trained on each item of the VA questionnaire and how to ask the questions correctly. New field workers are also mentored by experienced staff who have collected VAs for many years. This has ensured consistency and accuracy in the collection of VA data. A grieving period of 40 days is usually allowed for relatives to mourn the deceased before a VA is conducted, in line with customs and tradition. After this period, a field worker visits the household and enquires about the child. When the next of kin confirms that the child has died, the field worker proceeds to seek consent for the conduct of the VA. The VA interview is conducted in a culturally sensitive and ethically appropriate manner. Field workers are trained to be sympathetic and allow time for respondents to express their emotions and empathise with them.

### Determination of causes of death

VAs are collected by HDSS field workers using the Research Electronic Data Capture (REDCap) system. The forms are uploaded onto an online platform. Subsequently, two independent clinicians engage in an iterative process of reviewing VA forms and using the ICD, 10th Edition coding system to determine underlying and primary causes of death. In instances of diagnostic discrepancies, a consensus-based resolution process is employed, where the two clinicians meet to decide on a single cause of death. Within the context of the current PVS clinical trial, the determination of causes of death using VA is used to monitor the safety of the intervention.

The under-5 mortality surveillance approach in the two HDSSs has successfully identified and documented 1333 deaths from 1 September 2019 to 1 September 2023. VAs were carried out for 98.0% (1306) of these cases, and clinicians have coded 97.1% (1294) of them. A few VAs could not be conducted due to the migration of primary respondents from the HDSSs.

Of the 1294 coded deaths, comparable deaths occurred in females and males (659 vs 626, respectively, 9 with missing data). Most deaths occurred in the neonatal period (40.8%, n=528) and in those aged 1–11 months (25.7%, n=332). There were an equal number of deaths in children aged 12–23 months (16.8%, n=217) and 24–59 months (16.8%, n=217).

**Figure 3:**
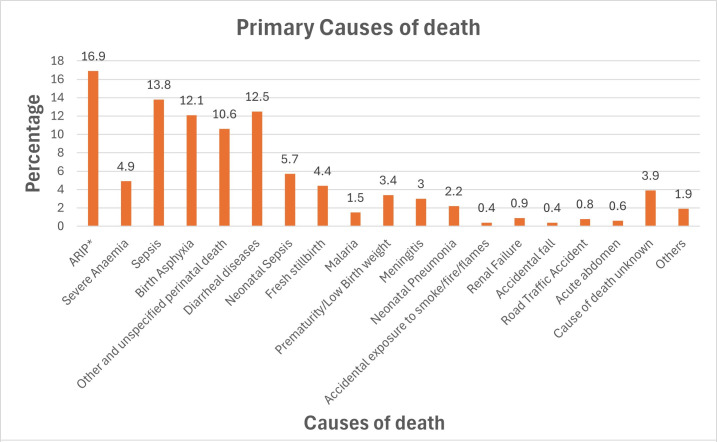
Primary causes of death determined using the physician-certified verbal autopsy method. *ARIP, acute respiratory infection including pneumonia.

The most common causes of death within the period are shown in figure 3. We found that the most common causes of death were acute respiratory infection including pneumonia (16.9%, n=219), sepsis (13.8%, n=179), diarrhoeal diseases (12.5%, n=162) and birth asphyxia (12.1%, n=156). A further breakdown of the deaths in the neonatal and post-neonatal periods is shown in online supplemental table 1.

10.1136/bmjgh-2023-014937.supp1 Supplementary data

## Lessons and challenges

The key challenges encountered in running the under-5 surveillance system have been summarised in table 1 and discussed below.

**Table 1:** Challenges and mitigation measures in undertaking under-5 mortality surveillance

Summary of challenges encountered in undertaking under-5 mortality surveillance and how they were mitigated		
**Broad challenges**	**Specific challenges**	**Mitigation measures**
Logistical challenges	Poor road network Risk of field workers being involved in accidents due to the poor road network Challenge in financing the activities of the HDSSs	Comprehensive motorbike training for new field workers to enable them to navigate the terrain Employing dedicated mechanics to maintain and repair motorbikes Ensuring field workers follow safety requirements when riding motorbikes HDSS activities are supported by core funding to MRCG at LSHTM from the UK Medical Research Council Encouraging projects to finance the activities of the HDSSs (the PVS Study currently funds the Fuladu West HDSS)
Household visit/data collection challenges	Challenge in transitioning from paper-based system to Electronic Data Capture (EDC) Meeting expectations of households during visits	Investing in training and retraining field workers on the EDC system Community engagement and sensitisation activities Emphasising the long-term benefits of collecting health and demographic data for the entire population during household visits Field workers take proactive steps to address immediate health needs by linking household members with project nurses and clinicians when necessary
HDSS setup challenges	Challenge in ensuring complete enumeration of the HDSSs during initial setup	The employment of field workers who deeply understand the local terrain, culture and community dynamics The involvement of village chiefs
Other challenges	Lack of integrating of HDSS data into national Civil Registration and Vital Statistics (CRVS) system	Future plans to engage the national CRVS bureau Encouraging government to take interest in HDSS data and to possibly consider financing them

HDSSs, Health and Demographic Surveillance Systems; MRCG at LSHTM, Medical Research Council Unit The Gambia at the London School of Hygiene and Tropical Medicine; PVS, Pneumococcal Vaccine Schedules.

An initial challenge during the setup of the HDSSs was ensuring that all villages and hamlets within the catchment area were enumerated. The employment of field workers who deeply understood the local terrain, culture and community dynamics played a role in navigating this challenge. The involvement of village chiefs also ensured accuracy in determining the names and locations of villages and hamlets. Also, until recently, most roads in the two HDSSs were untarred. The condition of untarred roads, particularly during the rainy season, posed significant difficulties for field workers using motorbikes to navigate the terrain. This caused practical difficulties in reaching households to collect data and posed safety risks. The solution to this challenge involved comprehensive motorbike training for new field workers, periodical refresher courses, and employing dedicated mechanics to maintain and repair the motorbikes. Additionally, to ensure their safety, field workers are obliged to use their helmets and protective gear when riding.

Additionally, from the inception of the Basse HDSS, paper forms were used to capture data. In 2016, Electronic Data Capture (EDC) was introduced.^25^ The shift from paper forms to EDC addressed several challenges associated with the traditional paper-based system, such as printing, errors in filling forms, data entry and storage. Although there were initial difficulties in adapting to the new electronic system, comprehensive training programmes enabled field workers to overcome these challenges. The EDC system not only reduced errors but also enhanced the overall quality of the collected data.

With a poverty rate of 64.6% in rural Gambia,^26^ some households expect some direct benefit whenever field workers visit them to collect data. However, community engagement and sensitisation activities have been vital in emphasising the long-term benefits of collecting health and demographic data for the entire population. Moreover, field workers take proactive steps to address immediate health needs by linking household members with project nurses and clinicians when necessary.

The Alkalos have been instrumental in ensuring the comprehensive capture of under-5 deaths. As respected community leaders, the Alkalos facilitate communication channels and foster trust, encouraging community members to provide accurate information about deaths. Engaging community members in a culturally sensitive manner helps to overcome potential barriers and ensures that deaths are reported comprehensively, contributing to the accuracy and inclusiveness of the approach. This collaborative approach has strengthened the overall effectiveness of death notification within the two HDSSs.

Another crucial lesson learnt from the implementation of the under-5 mortality surveillance is the significance of periodical training of field workers and diligent supervision of their work. The training extended beyond the technical aspects of mortality surveillance, encompassing essential soft skills for effective communication with community members and bereaved families. Clinicians who coded VAs also undertook quality control of the VA forms and instituted corrective actions whenever specific issues were identified. The feedback loop established between clinicians and field workers has proven instrumental in refining the quality of VAs. Moreover, each HDSS has several field supervisors who undertake field visits to address any practical challenges faced by field workers, maintain the quality of VAs and ensure adherence to protocols.

Though HDSSs provide vital longitudinal health and demographic data, they can be quite costly to set up and run. The Basse HDSS is maintained by core funding to the MRCG at LSHTM from the UK Medical Research Council, while the PVS Project maintains the Fuladu West HDSS. Without such external funding, it would be challenging to ensure the sustainability and robustness of HDSS activities. It may help if the Gambian government takes up the role of maintaining these HDSSs in the future. This will ensure national ownership and commitment and encourage the usage and consideration of HDSS data in formulating policies and allocating resources.

One area that must be improved is the integration of the under-5 mortality surveillance in the two HDSSs into the national CRVS system. Currently, no direct system is in place for integrating data collected within the two HDSSs at the national level. Even though such an endeavour can be complex, incorporating such data offers numerous benefits for the country.^27^ It will enhance the accuracy of vital statistics, enabling precise demographic analysis and aiding in public health planning and disease surveillance. In the future, it will be imperative to work out modalities to integrate mortality surveillance within the two HDSSs into the national CRVS system.

Also, though the PCVA method has been applied to successfully determine 1294 causes of under-5 deaths, it may become more difficult when this system is applied at a large scale, and many more deaths have to be coded. In such cases, automated computer algorithms such as InterVA, InSilicoVA and SmartVA can be employed to determine causes of death. Using such algorithms can also be cost-saving compared with the PCVA method. Additionally, more validation studies should be conducted across different settings to ensure consistency in cause of death determination using these algorithms.

## Conclusion

Without complete data on under-5 mortality, tracking progress towards achieving Sustainable Development Goal 3.2 will be difficult. Such data are also needed to ensure proper planning and prioritisation of scarce resources in LMICs. What has been described here is a model community-based under-5 mortality surveillance undertaken within two HDSSs in rural Gambia and strengthened by an ongoing clinical trial. The success of this simple system of mortality surveillance highlights the potential of training field workers with a minimum of secondary school education to undertake mortality surveillance in resource-limited settings. It also highlights the importance of close collaboration with communities in ensuring a successful system. Whole-scale replication in other settings may not be entirely feasible. However, the model can be adapted to other resource-limited settings to detect and determine the causes of death for children under-5.

## Data Availability

Data are available upon reasonable request. The mortality surveillance data that were used to determine the causes of death are available and could be obtained from the Health and Demographic Surveillance System Unit of the Medical Research Council Unit The Gambia at the London School of Hygiene and Tropical Medicine upon reasonable request.
