# Supplementary material for: Under-5 mortality surveillance in low-income and middle-income countries: insights from two Health and Demographic Surveillance Systems in rural Gambia
Source: BMJ Glob Health. 2024 Apr 2;9(4):e014937. doi: 10.1136/bmjgh-2023-014937 (PMC10989099; doi:10.1136/bmjgh-2023-014937)
Supplement: Supplementary data [file bmjgh-2023-014937supp001.pdf]

S1 Table. Primary causes of death

| Primary Cause of death | Frequency (N=1294) | Percentage (%) |
|---|---|---|
| **All deaths** | | |
| Acute respiratory infection including pneumonia (ARIP) | 219 | 16.9 |
| Severe Anaemia | 63 | 4.9 |
| Sepsis | 179 | 13.8 |
| Birth Asphyxia | 156 | 12.1 |
| Other and unspecified perinatal death | 137 | 10.6 |
| Diarrheal diseases | 162 | 12.5 |
| Neonatal Sepsis | 74 | 5.7 |
| Fresh stillbirth | 57 | 4.4 |
| Malaria | 19 | 1.5 |
| Prematurity/Low Birth weight | 44 | 3.4 |
| Meningitis | 39 | 3.0 |
| Neonatal Pneumonia | 29 | 2.2 |
| Accidental exposure to smoke, fire and flames | 5 | 0.4 |
| Renal Failure | 12 | 0.9 |
| Accidental fall | 5 | 0.4 |
| Road Traffic Accident | 10 | 0.8 |
| Acute abdomen | 8 | 0.6 |
| Cause of death unknown | 50 | 3.9 |
| Others | 25 | 1.9 |
| **Neonatal Period (0-27 days)** | | |
| Birth Asphyxia | 156 | 29.6 |
| Other and unspecified perinatal death | 137 | 26 |
| Neonatal Sepsis | 74 | 14 |
| Fresh stillbirth | 57 | 10.8 |
| Prematurity/Low Birth weight | 44 | 8.3 |
| Neonatal Pneumonia | 29 | 5.5 |
| Cause of death unknown | 13 | 2.5 |
| Severe Anaemia | 9 | 1.7 |
| Macerated stillbirth | 2 | 0.4 |
| Renal Failure | 2 | 0.4 |
| Others | 5 | 0.9 |
| **Post-neonatal period (1month-59months)** | | |
| Acute respiratory infection including pneumonia (ARIP) | 219 | 28.6 |
| Sepsis | 178 | 23.2 |
| Diarrheal diseases | 161 | 21 |

| | | |
|---|---|---|
| Severe Anaemia | 54 | 7.1 |
| Meningitis | 39 | 5.1 |
| Cause of death unknown | 37 | 4.8 |
| Malaria | 19 | 2.5 |
| Renal Failure | 10 | 1.3 |
| Road Traffic Accident | 10 | 1.3 |
| Acute abdomen | 7 | 0.9 |
| Accidental exposure to smoke, fire, flames | 5 | 0.7 |
| Accidental fall | 4 | 0.5 |
| Others | 23 | 3 |
